# Alteration of Prognostic Factors for Patients with Brain Metastases from Lung Cancer Before and After the Introduction of Immune Checkpoint Inhibitors: A Retrospective Single-Institution Study

**DOI:** 10.3390/cancers17183067

**Published:** 2025-09-19

**Authors:** Yohei Yamamoto, Tomona Maetani, Hiroki Narita, Yurika Terasawa, Naoki Kato, Yasuharu Akasaki, Yuichi Murayama, Toshihide Tanaka

**Affiliations:** 1 Department of Neurosurgery, Jikei University Daisan Hospital, 4-11-1 Izumi-Honcho, Komae, Tokyo 201-0003, Japan; yohei_1109@yahoo.co.jp (Y.Y.); torapon_10.05@icloud.com (T.M.); narikei592@gmail.com (H.N.); y.terasawa26@gmail.com (Y.T.); nao-kth@jikei.ac.jp (N.K.); 2 Department of Neurosurgery, The Jikei University School of Medicine, 3-25-8 Nishi-Shinbashi, Minato, Tokyo 105-8461, Japan; akasaki@jikei.ac.jp (Y.A.);

**Keywords:** brain metastases, immune checkpoint inhibitors, neutrophil-to-lymphocyte ratio

## Abstract

Background/Objectives: Immune checkpoint inhibitors (ICIs) have improved outcomes in advanced lung cancer, but their real-world impact on patients with brain metastases remains insufficiently characterized. This study aimed to compare treatment outcomes before and after the introduction of ICIs and to identify prognostic factors in patients with lung cancer brain metastases. Methods: We retrospectively analyzed 186 patients treated for brain metastases from lung cancer at our institution between 2014 and 2023. Patients were classified into a Pre-ICI group (N = 93, 2014–2018) and a Post-ICI group (N = 93, 2019–2023). Overall survival (OS) was analyzed by Kaplan–Meier method and Cox regression. Baseline factors included age, sex, histology, Charlson–Deyo score, extracranial metastases, radiotherapy, systemic therapy, and neutrophil-to-lymphocyte ratio (NLR, cutoff = 4). Results: Median OS improved significantly in the Post-ICI group compared with the Pre-ICI group (10.9 vs. 4.7 months, *p* < 0.01). When stratified by systemic therapy, median OS was 4.7 months with conventional chemotherapy, 14.7 months with molecular targeted therapy overall, further prolonged to 25.5 months in the Post-ICI era, and 23.4 months for all patients receiving ICIs. The most notable benefits were observed in patients with squamous cell carcinoma and small cell carcinoma. Patients with NLR ≥ 4 showed shorter OS, but NLR did not remain significant in multivariate analysis. In EGFR-mutant adenocarcinoma, the survival benefit from ICIs was limited. Conclusions: ICIs significantly improved survival in patients with lung cancer brain metastases, particularly those with squamous cell carcinoma or small cell carcinoma. NLR may provide supportive prognostic information, while molecular targeted therapy and ICIs represent major drivers of improved survival in this population.

## Simple Summary

Brain metastases are a common complication of lung cancer and often lead to poor prognosis. This study retrospectively analyzed the survival of lung cancer patients with brain metastases before and after the introduction of immune checkpoint inhibitors. We found that immune checkpoint inhibitors significantly improved survival in patients with squamous cell and small cell lung cancers. In contrast, the benefit of immune checkpoint inhibitors was limited in adenocarcinoma, likely due to the use of molecularly targeted therapies. In addition, the simple blood marker of neutrophil-to-lymphocyte ratio may help identify patients likely to benefit from immune checkpoint inhibitors.

## 1. Introduction

Lung cancer is a malignant tumor that frequently shows metastasis to the brain. Management of metastasis requires a combination of systemic and local therapies including surgery, radiotherapy (whole-brain radiotherapy [WBRT] or stereotactic radiosurgery [SRS]), and chemotherapy. Recent advances in immune checkpoint inhibitors (ICIs) have dramatically improved outcomes for various solid tumors, including non-small cell lung cancer (NSCLC). Despite these developments, real-world evidence regarding the efficacy of ICIs and their prognostic factors in lung cancer patients with brain metastases (BM) remains limited. Although programmed death-ligand 1 (PD-L1), tumor mutation burden (TMB), and microsatellite instability [1] are known as predictive biomarkers for therapy with ICIs, background factors such as age, Karnofsky performance scale (KPS), control for extracranial metastases, and histological subtypes may to some extent predict clinical outcomes for patients with BM from primary cancers.

Given the mechanism of effectiveness and algorithm of therapeutic strategy, predictive biomarkers for ICIs therapy should be required based on the immunological tumor microenvironment and molecular pathological features.

As a potential predictive biomarker for ICIs therapy, neutrophil-to-lymphocyte ratio (NLR) is a peripheral blood biomarker that has received attention as an indicator of systemic inflammation and the tumor immune milieu. While NLR has been reported as a prognostic factor in various NSCLC settings, its clinical utility in patients with BM from lung cancer remains unclear.

In this study, we compared the clinical characteristics and treatment outcomes of patients with BM from lung cancer before and after the introduction of ICIs, with a particular focus on the prognostic value of NLR.

## 2. Materials and Methods

This retrospective single-institution study aimed to elucidate the impact of the introduction of ICIs on treatment outcomes and prognostic factors in patients with BM from lung cancer. Particular focus was given to NLR as a clinically accessible marker of inflammation.

Participants comprised 186 patients diagnosed and treated for lung cancer BM at our institution between 2014 and 2023. Patients were divided into a Pre-ICIs group (n = 93, treated in 2014–2018) and a Post-ICIs group (n = 93, treated in 2019–2023). Baseline characteristics, treatment modalities, and median overall survival (mOS) were retrospectively compared. Baseline factors analyzed included age, sex, histological subtype (adenocarcinoma, squamous cell carcinoma, small cell carcinoma:SCLC, etc.), control of the primary lesion, number of BM, presence of extracranial metastases, and intervention with radiotherapy (including WBRT or SRS) and chemotherapy (including molecularly targeted agents and ICIs). ICI indication was determined according to the diagnostic resources available during the study period. Before 2019, companion diagnostics for EGFR and ALK alterations were routinely performed. After ICI approval in Japan, PD-L1 immunohistochemistry was applied to guide treatment selection. From 2020, we introduced the OncoPanel Dx Target Test (a next-generation sequencing–based companion diagnostic in Japan) in selected cases; however, this platform does not calculate or report tumor mutational burden (TMB). In addition, under the Japanese clinical practice guidelines, TMB testing is not required for determining ICI eligibility in lung cancer. Consequently, TMB values were not available in this cohort and did not contribute to treatment selection.

Chemotherapy categories (conventional chemotherapy, molecular targeted therapy, or ICIs) were defined based on systemic therapies initiated after the diagnosis of brain metastases. Patients who had previously received ICIs during earlier treatment phases for their primary lung cancer but were subsequently managed with other systemic therapies or best supportive care after the onset of brain metastases were classified according to the treatment administered after brain metastasis. This approach was applied consistently across histological subtypes, including small cell carcinoma (SCLC) and squamous cell carcinoma. In these subtypes, the relatively low number of patients recorded as receiving ICIs reflects our strict classification method, which was based only on systemic therapies administered after the diagnosis of brain metastases. Consequently, patients who had received ICIs during earlier treatment phases for their primary lung cancer but were subsequently managed with other systemic therapies or best supportive care after brain metastasis were not counted in the ICI group. In addition, clinical factors such as poor performance status, rapid disease progression, and the gradual implementation of institutional protocols at the beginning of the study period also contributed to the lower apparent rate of ICI use in these subgroups.

NLR ≥ 4 has been significantly associated with poorer prognosis in patients with lung cancer following the administration of ICIs [2]. As a result, NLR before treatment with ICIs was additionally used to dichotomize patients (using a cutoff of 4) for comparison of mOS.

Survival analyses were conducted using the Kaplan–Meier method, and multivariate analysis was performed using Cox proportional hazards modeling. Baseline factors were compared between groups using one-way analysis of variance (ANOVA). All statistical analyses were conducted using R software (version 4.3.0; R Foundation for Statistical Computing, Vienna, Austria), with the significance level set at *p* < 0.05.

The complete patient-level dataset used for all statistical analyses is provided as Supplementary Table S1 to ensure transparency and reproducibility. This dataset includes all enrolled patients’ baseline characteristics, treatment modalities, and survival outcomes.

The institutional review board approved this study (approval no. 33-256). Due to the retrospective nature of the investigation, the requirement for informed consent was waived.

## 3. Results

The Pre-ICIs and Post-ICIs groups showed no significant differences in pretreatment baseline characteristics, such as age, sex, histological subtype, number of brain metastases, presence of extracranial metastases, or performance status. However, regarding treatment-related factors, the Post-ICIs group exhibited significant changes compared to the Pre-ICIs group: the use of conventional chemotherapy decreased, while the use of ICIs increased; the use of whole-brain radiotherapy decreased, whereas stereotactic radiosurgery increased; and the proportion of neurological deaths was also higher in the Post-ICIs group (Table 1).

**Figure 1:**
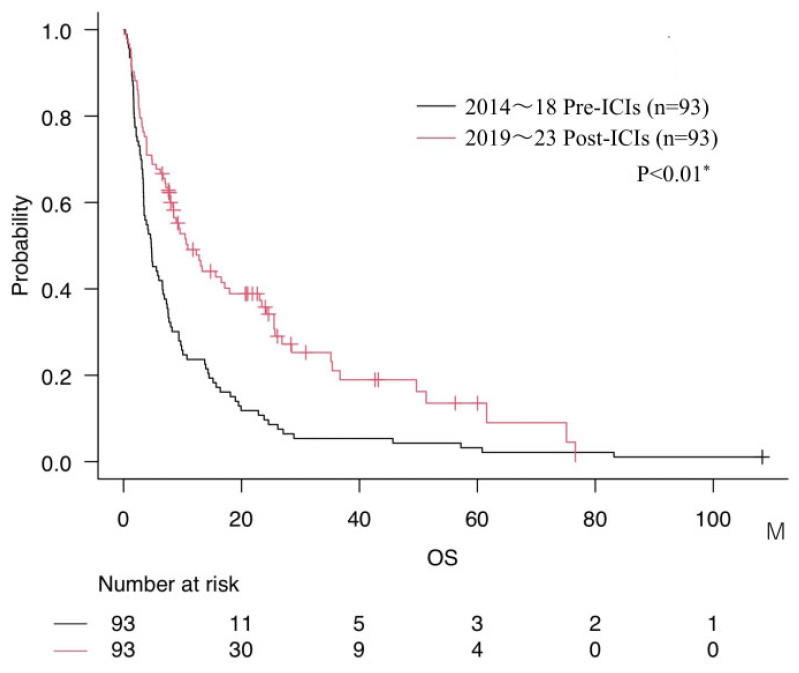
Comparison of overall survival (mOS) between Pre-ICIs and Post-ICIs groups. * = *p* < 0.05.

Compared with the Pre-ICIs group (mOS 4.7 months), the Post-ICIs group demonstrated significantly longer survival (mOS 10.9 months, *p* < 0.01). (Figure 1). Furthermore, when stratified by treatment modality, the median OS was 4.7 months in patients receiving conventional chemotherapy, 14.7 months for those treated with molecular targeted therapy overall, which was further prolonged to 25.5 months when limited to the Post-ICIs era, and 23.4 months for those treated with ICIs.

**Figure 2:**
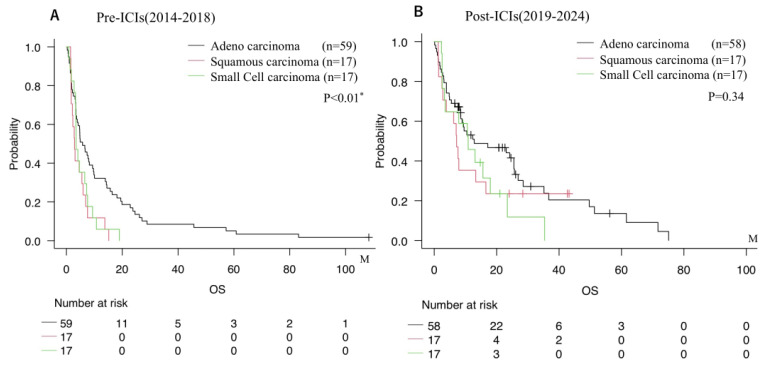
Overall survival stratified by histological subtype (squamous cell carcinoma, adenocarcinoma, small cell carcinoma, etc.). (**A**) Pre-ICIs era (2014–2018). (**B**) Post-ICIs era (2019–2023). * = *p* < 0.05.

Notably, comparison of histological subtypes across treatment eras revealed distinct survival trends (Figure 2). In the Pre-ICI group, patients with adenocarcinoma exhibited the most favorable prognosis among all subtypes. However, following the introduction of ICIs, survival of patients with small cell carcinoma or squamous cell carcinoma improved markedly, narrowing the survival gap between these subtypes and adenocarcinoma in the Post-ICIs era.

**Figure 3:**
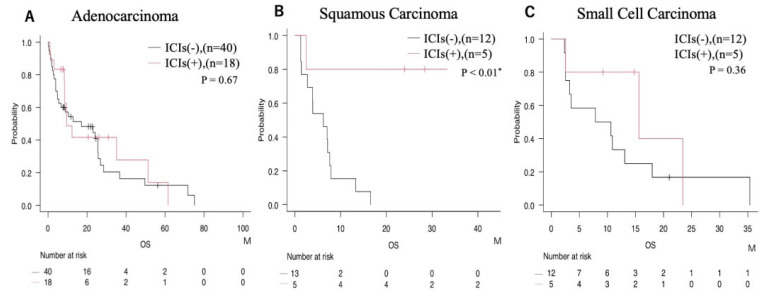
Prognostic comparison according to ICIs administration status within each histological subtype. (**A**) Adenocarcinoma; (**B**) squamous carcinoma; (**C**) small cell carcinoma. * = *p* < 0.05.

The impact of ICIs on prognosis for each histological subtype was thoroughly evaluated. In cases of adenocarcinoma, no significant difference in survival was observed between patients who did and did not receive ICIs (Figure 3A). Conversely, in squamous cell carcinoma, use of ICIs was associated with a significant increase in overall survival (Figure 3B). For small cell carcinoma, the level of statistical significance was not reached, but a trend was seen toward improved survival with ICI therapy (Figure 3C).

**Figure 4:**
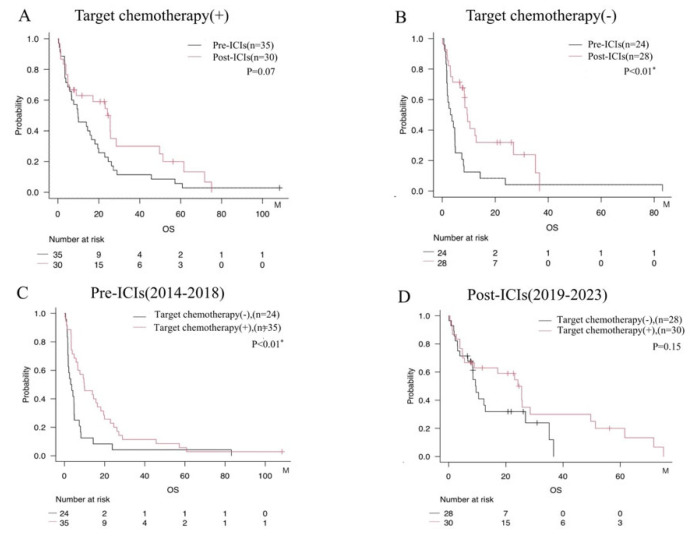
Survival analysis evaluating the impact of ICIs administration stratified by use of molecularly targeted agents among patients with adenocarcinoma. (**A**) Comparison by era among patients treated with molecularly targeted agents. (**B**) Comparison by era among patients not treated with molecularly targeted agents. (**C**) Comparison between patients with and without treatment using molecularly targeted agents in the Pre-ICIs era. (**D**) Comparison between patients with and without molecularly targeted agents in the Post-ICIs era. * = *p* < 0.05.

We focused exclusively on adenocarcinoma cases and examined the impact of ICIs based on the use of molecularly targeted agents (Figure 4A,B). The results indicate that the prognostic benefit of ICIs in adenocarcinoma was limited to patients for whom molecularly targeted therapy was not appropriate (Figure 4A,B). Further, even among patients receiving molecularly targeted agents, a trend was seen toward improved survival over time, reflecting advances in therapy in the Post-ICI era (Figure 4C,D).

**Figure 5:**
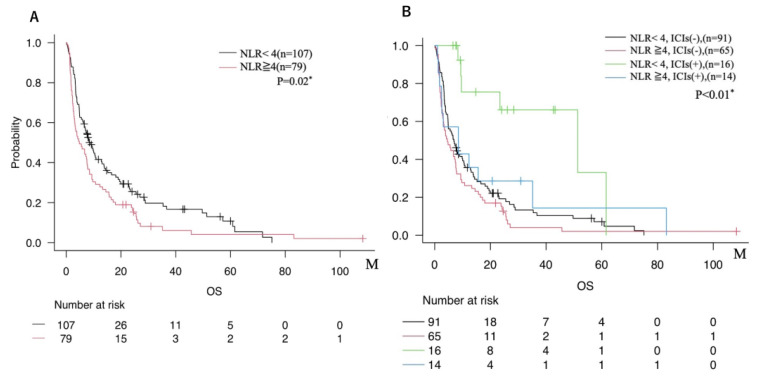
(**A**) Comparison of overall survival between two groups dichotomized by NLR cutoff (NLR < 4 vs. NLR ≥ 4). (**B**) Overall survival among four subgroups based on both NLR (cutoff 4) and ICIs administration status (NLR < 4 with ICIs use; NLR < 4 without ICIs use; NLR ≥ 4 with ICIs use; NLR ≥ 4 without ICIs use). * = *p* < 0.05.

When patients were divided into two groups based on the cutoff value of NLR = 4, those with NLR ≥ 4 tended to show shorter mOS than those with NLR < 4 (Figure 5A). In addition, subgroup analyses stratified by both NLR and ICIs use showed that patients with NLR < 4 who received ICIs exhibited the most favorable prognosis. In contrast, those with NLR ≥ 4 who did not receive ICIs had the poorest prognosis (Figure 5B).

Multivariate analysis (Table 2) identified various factors as independent predictors of better overall survival. In the Pre-ICIs group, higher KPS, surgical intervention, SRS, and use of molecularly targeted agents were all linked to improved prognosis. In the Post-ICIs group, higher KPS, surgical intervention, use of molecularly targeted agents, solitary BM, and administration of ICIs emerged as significant favorable prognostic factors. In contrast, NLR was not maintained as an independent prognostic factor.

Furthermore, because small cell carcinoma is often analyzed separately from NSCLC, we performed additional analyses excluding these cases. The impact on overall survival was limited, with the median OS changing only slightly (Pre-ICIs: 4.7 to 4.8 months; Post-ICIs: 10.9 to 10.4 months). Baseline characteristics also showed minimal changes, except that the previously observed higher proportion of neurological deaths in the Post-ICIs group lost statistical significance. Similarly, the association between NLR and survival outcomes remained essentially unchanged (Supplemental Tables S2 and S3, Supplemental Figures S1 and S2).

## 4. Discussion

This study demonstrated that the introduction of ICIs contributed to improved median overall survival (mOS) in patients with brain metastases (BM) from lung cancer, with particularly notable benefits observed in those with squamous cell carcinoma or small cell lung cancer. This finding is consistent with previous analyses using large national databases [3]. A nationwide US study by Takamori et al. [3] using propensity score matching similarly showed that ICI therapy extended survival in patients with BM (mOS 12.8 months vs. 10.1 months). Despite differences in cohort size and clinical setting, both studies consistently demonstrate the survival benefit of ICIs in this population.

An extensive retrospective analysis across multiple cancer types further reported that the neutrophil-to-lymphocyte ratio (NLR) correlates with ICI response and mOS, particularly when combined with tumor mutational burden (TMB) for patient stratification [4].

The efficacy of ICIs in the central nervous system (CNS) is influenced by the blood–brain barrier (BBB). Although the BBB limits the penetration of large molecules such as ICIs, stereotactic radiosurgery (SRS) can transiently disrupt the BBB and may potentiate immunotherapy; the abscopal effect provides an additional biological rationale for combination approaches [5,6,7]. Combining SRS with ICIs has been clinically associated with improved local control and mOS in NSCLC patients with BM [8], and enhanced intracranial control has been observed in melanoma BM treated with CTLA-4 inhibitors plus SRS [9]. Moreover, real-world practice has seen increased upfront SRS alongside the introduction of ICIs, correlating with better intracranial progression-free survival [10]. Considering these factors, the timing of onset—whether the primary lesion and brain metastasis occur synchronously or metachronously—substantially influences treatment strategy. Metachronous cases are often affected by prior therapies, whereas synchronous cases allow for more straightforward planning of ICIs administration in conjunction with radiotherapy. However, such situations also demand close collaboration with other specialties, particularly in determining treatment priorities between intracranial and primary lesions. In this context, neurosurgeons play a critical role by promptly controlling intracranial disease, thereby enabling other teams to focus on treating the primary lesion and ensuring a seamless continuum of care.

Corticosteroids are often required for edema and symptom control in BM, yet may attenuate ICI activity. High-dose steroids have been linked to reduced efficacy of PD-1 inhibitors [6,11], and concomitant steroid use can weaken ICI effects, particularly in patients with high inflammatory burden such as elevated NLR [12]. In our cohort, however, OS did not differ between steroid-treated and non-steroid-treated patients during ICI therapy. Only 2 of 20 patients were on steroids at baseline (prior to ICI initiation), limiting statistical power; although prior reports suggest baseline steroid exposure may compromise ICI efficacy [12], our data did not confirm this effect within the sample size available.

For adenocarcinoma, many patients harboring EGFR mutations appropriately received molecularly targeted therapy as first-line treatment, which can limit the incremental benefit of ICIs. A meta-analysis reported inferior efficacy of PD-1/PD-L1 inhibitors in EGFR-mutant NSCLC compared with EGFR-wildtype disease [5], consistent with our findings (Figure 3); similarly, reduced ICI responsiveness has been reported in tumors with EGFR mutations or ALK rearrangements [13]. Conversely, pembrolizumab combined with chemotherapy has proven both safe and effective in NSCLC patients with BM [14]. Large-scale analyses also show that the introduction and widespread use of newer EGFR-TKIs significantly improved survival irrespective of BM status [15], and the development of next-generation targeted agents (ALK, ROS1, RET) with superior CNS penetration has further expanded options and prolonged intracranial control [16,17].

In our cohort, survival stratified by treatment modality highlights this landscape shift: conventional chemotherapy (mOS 4.7 months), molecular targeted therapy overall (mOS 14.7 months), molecular targeted therapy limited to the Post-ICI era (mOS 25.5 months), and ICIs pooled across eras (mOS 23.4 months). These patterns likely reflect both the uptake of next-generation TKIs with improved CNS activity after 2019 and guideline-concordant expansion of ICI use. In selected EGFR-mutant cases, sequential administration of TKIs following ICIs has shown potential benefit [18]. Together with evidence that higher TMB is associated with greater sensitivity to PD-1 inhibitors [19], these observations support integrated stratification using PD-L1, TMB, and NLR.

NLR is increasingly recognized as a practical, non-invasive biomarker of systemic inflammation that reflects the tumor immune milieu [20]. Although the optimal cutoff remains debated—thresholds of 4 and 5 have both been reported in metastatic settings [21]—our sensitivity analyses produced similar trends with either threshold, and NLR = 4 provided the clearest survival discrimination; hence, it was adopted in this study. Patients with NLR < 4 who received ICIs exhibited the most favorable prognosis, whereas those with NLR ≥ 4 without ICIs had the poorest outcomes, suggesting an interaction between inflammatory status and immunotherapy benefit.

From an immunopathological perspective, elevated neutrophils are linked to expansion of myeloid-derived suppressor cells (MDSCs), which promote angiogenesis and inhibit cytotoxic T-cell activity, thereby establishing an immunosuppressive milieu; low lymphocyte counts indicate impaired adaptive immunity and reduced CD8+ T-cell function—both mechanisms plausibly diminishing ICI effectiveness [22]. Consistent with this biology, large meta-analyses and multicenter cohorts have shown that high NLR predicts poorer survival among ICI-treated patients across tumor types, including NSCLC [20,23,24,25]. By contrast, while NLR has been examined with targeted therapies (e.g., EGFR-TKIs), its predictive value appears more robust in the context of immunotherapy [26,27,28]. Overall, these data support using NLR to refine selection of patients most likely to benefit from ICIs.

Because small cell carcinoma is often analyzed separately in lung cancer studies, we performed supplementary analyses excluding SCLC. The median OS of the Pre- and Post-ICI groups remained essentially unchanged, and the association between NLR and prognosis was preserved (Supplementary Tables S2 and S3; Supplementary Figures S1 and S2). Thus, despite distinct biology and therapeutics, our principal conclusions regarding ICIs and NLR were robust regardless of SCLC inclusion. Notably, ICIs improved survival particularly in squamous cell carcinoma and SCLC (Figure 3). In SCLC, accumulating evidence suggests that prophylactic cranial irradiation and multimodal strategies—including combination radiotherapy with ICIs—may be promising [29,30].

Recent innovations such as osimertinib and sunvozertinib have further extended survival in EGFR-mutant lung cancer; improved CNS penetration has translated into better control of brain and leptomeningeal metastases [31,32,33,34,35,36,37]. Against this backdrop, our cohort illustrates how systemic advances reshape neurological outcomes: among deceased patients, neurological deaths accounted for 10 of 92 deaths (10.8%) in the Pre-ICI era versus 16 of 69 (23.2%) in the Post-ICI era (*p* = 0.04). Nevertheless, OS among patients who ultimately died from neurological causes improved from 1.7 to 3.3 months, and OS in carcinomatous meningitis increased from 2.8 to 4.9 months, indicating that modern systemic therapies can meaningfully extend survival even in traditionally dismal scenarios.

For neurosurgeons, these trends reduce the frequency of survival-prolonging resections but reinforce an essential role in: (i) palliative procedures such as cerebrospinal fluid diversion for hydrocephalus or decompression for symptomatic relief; (ii) obtaining diagnostic tissue, especially in synchronous presentations; and (iii) managing intracranial disease refractory to systemic therapy. Thus, as systemic therapies increasingly shape prognosis, neurosurgical practice must adapt and integrate within multidisciplinary care to optimize both survival and neurological quality of life.

In summary, even within the constraints of a single-institution retrospective cohort, our findings show that ICIs significantly improve survival in lung cancer patients with BM, and that NLR serves as a simple, clinically actionable biomarker to stratify benefit. These conclusions hold whether or not SCLC cases are included in the analysis, and they delineate a continuing, indispensable role for neurosurgeons in the ICI era.

In recent years, the therapeutic landscape of lung cancer with brain metastases has undergone a paradigm shift with the advent of immune checkpoint inhibitors (ICIs) and the development of molecular targeted agents with improved central nervous system (CNS) penetration. These advances have changed not only overall survival outcomes but also the clinical course of neurological complications. In our cohort, analyses focusing on deceased patients revealed that neurological deaths accounted for 10 of 92 deaths (10.8%) in the Pre-ICI era and 16 of 69 deaths (23.2%) in the Post-ICI era (*p* = 0.04). Nevertheless, additional analyses showed that median OS among patients who eventually died from neurological causes improved from 1.7 to 3.3 months, and in carcinomatous meningitis, which frequently represents the terminal pathway of neurological death, OS increased from 2.8 to 4.9 months. These findings indicate that, even in conditions traditionally associated with dismal prognosis, modern systemic therapies can provide meaningful survival extension.

For neurosurgeons, these changes underscore a transformation in the nature of clinical involvement. In the Pre-ICI era, neurosurgeons frequently played a leading role in the management of brain metastases through surgical resection or other local interventions, as intracranial disease often dictated the clinical course. By contrast, in the Post-ICI era, systemic disease control has improved, and ICIs or targeted therapies may render previously symptomatic or unresectable lesions stable or asymptomatic. As a result, the frequency of direct neurosurgical intervention for survival prolongation may decline, yet the clinical responsibilities of neurosurgeons remain essential. Neurosurgeons continue to play a critical role in several domains: (i) palliative interventions such as cerebrospinal fluid diversion for hydrocephalus or decompression in symptomatic cases; (ii) obtaining diagnostic tissue, particularly in cases with synchronous detection of primary and metastatic lesions; and (iii) managing intracranial lesions that remain refractory to systemic therapy.

Thus, while systemic therapies increasingly shape the prognosis of lung cancer patients with brain metastases, neurosurgical practice must adapt to complement these paradigm shifts. The integration of neurosurgical expertise with systemic therapies is likely to become even more important, as collaborative multidisciplinary strategies are required to optimize both survival outcomes and neurological quality of life.

There are several limitations to this study. This single-institution, retrospective analysis with a modest sample size is subject to selection bias and unmeasured confounding. Treatment exposures were not uniform across the cohort—including ICI agent, radiotherapy timing, and steroid use—and the limited adoption of ICIs in SCLC reflects regulatory and implementation timing in Japan rather than therapeutic intent. Biomarker data (e.g., TMB and comprehensive genomic profiling) were also incomplete. Finally, cases treated between 2019 and 2023 had inherently shorter follow-up, possibly conservatively influencing survival estimates. Despite these constraints, the observed survival extension after ICI introduction and the clinical utility of NLR-based stratification were consistent across analyses and remain the key messages of this study.

## 5. Conclusions

This study demonstrates that the introduction of ICIs has contributed to improved survival in patients with BM from lung cancer, with particularly pronounced benefits observed in those with squamous cell carcinoma and small cell lung cancer. Furthermore, clinical background factors such as NLR, histological subtype, and prior treatment history were shown to influence ICIs’ efficacy, underscoring the need for treatment stratification in clinical practice. These findings highlight that even in this challenging patient population, meaningful survival extension can be achieved by tailoring therapy, and the integration of radiotherapy, careful steroid management, and novel molecular targeted agents may further optimize outcomes. At the same time, neurosurgeons remain integral in ensuring diagnostic accuracy, local disease control, and supportive interventions in this evolving treatment era.

## Figures and Tables

**Table 1:** Clinical characteristics of PreICIs (2014–2018) and PostICIs (2019–2023).

	Main Factor	Sub Factor	PreICIs	PostICIs	*p* Value
Pretreatment factors	age	over 70	54	65	0.13
		under 70	39	28	
	sex	female	30	40	0.13
		male	63	53	
	symptomatology	asymptomatic	49	51	0.77
		symptomatic	44	42	
	KPS	under 60	49	38	0.11
		over 70	44	55	
	cyst lesion	(−)	67	63	0.53
		(+)	26	30	
	hematoma	(−)	91	92	0.56
		(+)	2	1	
	meningitis	(−)	87	80	0.1
		(+)	6	13	
	extra cranial metastasis	(−)	53	55	0.77
		(+)	40	38	
	Charlson-Deyo score	0–1	65	72	0.32
		over 2	28	21	
	Number of metastatic lesions	multiple	64	56	0.61
		single	29	37	
	NLR < 4	(−)	49	58	0.23
		(+)	44	35	
Therapeutic factors	surgery	(−)	84	79	0.27
		(+)	9	14	
	chemotherapy	(−)	15	14	0.84
		(+)	78	79	
	conventional chemotherapy	(−)	51	72	<0.01 *
		(+)	42	21	
	Molecular targeted therapy	(−)	56	60	0.55
		(+)	37	33	
	ICIs	(−)	91	65	<0.01 *
		(+)	2	28	
	radiation	(−)	25	28	0.63
		(+)	68	65	
	SRS	(−)	64	43	<0.01 *
		(+)	29	50	
	whole brain radiation	(−)	50	76	<0.01 *
		(+)	43	17	
	neuro death	(−)	82	53	0.04 *
		(+)	10	16	

ICI = immune checkpoint inhibitor, KPS = Karnofsky performance status; NLR = Neutrophil/Lymphocyte ratio, SRS = stereotactic radiosurgery, * = *p* < 0.05.

**Table 2:** Multivariate analysis of median survival times for brain metastasis patients treated in pre-ICIs and post-ICIs.

PreICIs				PostICIs			
Factor	HR	95%CI	*p* Value	Factor	HR	95%CI	*p* Value
KPS over70	0.53	0.33–0.87	0.01 *	KPS over70	0.31	0.18–0.54	<0.01 *
surgery	0.44	0.20–0.97	0.04 *	single lesion	0.36	0.20–0.65	<0.01 *
SRS	0.55	0.33–0.93	0.03 *	surgery	0.27	0.12–0.61	<0.01 *
Target chemotherapy	0.55	0.34–0.89	0.01 *	Target chemotherapy	0.33	0.17–0.64	<0.01 *
				ICIs	0.37	0.19–0.70	<0.01 *

CI = confidence interval, HR = hazard ratio, ICIs = immune checkpoint inhibitor, KPS = karnofsky performance status, SRS = stereotactic radiosurgery, * = *p* < 0.05.

## Data Availability

The original contributions presented in this study are included in the article/Supplementary Materials. Further inquiries can be directed to the corresponding author.

## Supplementary Materials

The following supporting information can be downloaded at: https://www.mdpi.com/article/10.3390/cancers17183067/s1, Table S1: Patient-level dataset used for all analyses; Table S2: Baseline characteristics and outcomes excluding small cell lung carcinoma (SCLC); Table S3: Multivariable Cox analysis excluding SCLC; Figure S1: Kaplan–Meier overall survival comparing Pre-ICIs (2014–2018) vs. Post-ICIs (2019–2023) after excluding SCLC; Figure S2: Overall survival stratified by NLR (cutoff 4) and ICI use after excluding SCLC.

## Abbreviations

The following abbreviations are used in this manuscript:

ALK	Anaplastic lymphoma kinase
ANOVA	Analysis of variance
BBB	Blood-brain barrier
BM	Brain metastases
EGFR	Epidermal growth factor receptor
ICIs	Immune checkpoint inhibitors
KPS	Karnofsky performance scale
mOS	Median overall survival
NA	Not available
OS	Overall survival
PD-1	Programmed cell death 1
PD-L1	Programmed death-ligand 1
RET	Rearranged during Transfection
ROS1	c-ros oncogene 1, receptor tyrosine kinase
SD	Standard deviation
SRS	Stereotactic radiosurgery
SRT	Stereotactic radiation therapy
TKIs	Tyrosine kinase inhibitors

## References

[1] Ranjan T., Podder V., Margolin K., Velcheti V., Maharaj A., Ahluwalia M.S. (2024). Immune Checkpoint Inhibitors in the Management of Brain Metastases from Non-Small Cell Lung Cancer: A Comprehensive Review of Current Trials, Guidelines and Future Directions. Cancers..

[2] Yu Y., Qian L., Cui J. (2017). Value of neutrophil-to-lymphocyte ratio for predicting lung cancer prognosis: A meta-analysis of 7,219 patients. Mol. Clin. Oncol..

[3] Takamori S., Komiya T., Powell E. (2021). Survival benefit from immunocheckpoint inhibitors in stage IV non-small cell lung cancer patients with brain metastases: A National Cancer Database propensity-matched analysis. Cancer Med..

[4] Valero C., Lee M., Hoen D., Weiss K., Kelly D.W., Adusumilli P.S., Paik P.K., Plitas G., Ladanyi M., Postow M.A. (2021). Pretreatment neutrophil-to-lymphocyte ratio and mutational burden as biomarkers of tumor response to immune checkpoint inhibitors. Nat. Commun..

[5] Ngwa W., Irabor O.C., Schoenfeld J.D., Hesser J., Demaria S., Formenti S.C. (2018). Using immunotherapy to boost the abscopal effect. Nat. Rev. Cancer..

[6] Pangal D.J., Yarovinsky B., Cardinal T., Cote D.J., Ruzevick J., Attenello F.J., Chang E.L., Ye J., Neman J., Chow F. (2022). The abscopal effect: Systematic review in patients with brain and spine metastases. Neurooncol Adv..

[7] Lee C.K., Man J., Lord S., Links M., Gebski V., Mok T., Yang J.C. (2017). Checkpoint Inhibitors in Metastatic EGFR-Mutated Non-Small Cell Lung Cancer-A Meta-Analysis. J. Thorac. Oncol..

[8] Chen S., Yu Q., Jiang W., Lu Y., Zhao Y., Wang H. (2023). Rechallenge with EGFR-TKI after failure of immunotherapy is considered an effective treatment for advanced lung adenocarcinoma patients with EGFR exon 19 deletion: A case report. Front Med..

[9] Abdulhaleem M., Johnston H., D’Agostino R., Lanier C., LeCompte M., Cramer C.K., Ruiz J., Lycan T., Lo H.W., Watabe K. (2022). Local control outcomes for combination of stereotactic radiosurgery and immunotherapy for non-small cell lung cancer brain metastases. J. Neurooncol..

[10] Wasilewski D., Radke J., Xu R., Raspe M., Trelinska-Finger A., Rosenstock T., Poeser P., Schumann E., Lindner J., Heppner F. (2022). Effectiveness of Immune Checkpoint Inhibition vs Chemotherapy in Combination with Radiation Therapy Among Patients with Non-Small Cell Lung Cancer and Brain Metastasis Undergoing Neurosurgical Resection. JAMA Netw. Open.

[11] Rizvi N.A., Hellmann M.D., Snyder A., Kvistborg P., Makarov V., Havel J.J., Lee W., Yuan J., Wong P., Ho T.S. (2015). Cancer immunology. Mutational landscape determines sensitivity to PD-1 blockade in non-small cell lung cancer. Science.

[12] Lauko A., Thapa B., Sharma M., Muhsen B., Barnett A., Rauf Y., Borghei-Razavi H., Tatineni V., Patil P., Mohammadi A. (2021). Neutrophil to lymphocyte ratio influences impact of steroids on efficacy of immune checkpoint inhibitors in lung cancer brain metastases. Sci. Rep..

[13] Gainor J.F., Shaw A.T., Sequist L.V., Fu X., Azzoli C.G., Piotrowska Z., Huynh T.G., Zhao L., Fulton L., Schultz K.R. (2016). EGFR Mutations and ALK Rearrangements Are Associated with Low Response Rates to PD-1 Pathway Blockade in Non-Small Cell Lung Cancer: A Retrospective Analysis. Clin. Cancer Res..

[14] Goldberg S.B., Schalper K.A., Gettinger S.N., Mahajan A., Herbst R.S., Chiang A.C., Lilenbaum R., Wilson F.H., Omay S.B., Yu J.B. (2020). Pembrolizumab for management of patients with NSCLC and brain metastases: Long-term results and biomarker analysis from a non-randomised, open-label, phase 2 trial. Lancet Oncol..

[15] Yamaguchi H., Wakuda K., Fukuda M., Kenmotsu H., Mukae H., Ito K., Chibana K., Inoue K., Miura S., Tanaka K. (2021). A Phase II Study of Osimertinib for Radiotherapy-Naive Central Nervous System Metastasis From NSCLC: Results for the T790M Cohort of the OCEAN Study (LOGIK1603/WJOG9116L). J. Thorac. Oncol..

[16] de Jong L.A.W., Sparidans R.W., van den Heuvel M.M. (2023). Cerebrospinal Fluid Concentration of the RET Inhibitor Pralsetinib: A Case Report. Case Rep. Oncol..

[17] Gil M., Knetki-Wróblewska M., Niziński P., Strzemski M., Krawczyk P. (2023). Effectiveness of ALK inhibitors in treatment of CNS metastases in NSCLC patients. Ann. Med..

[18] An Y., Jiang W., Kim B.Y.S., Qian J.M., Tang C., Fang P., Logan J., D’Souza N.M., Haydu L.E., Wang X.A. (2017). Stereotactic radiosurgery of early melanoma brain metastases after initiation of anti-CTLA-4 treatment is associated with improved intracranial control. Radiother. Oncol..

[19] Arbour K.C., Mezquita L., Long N., Rizvi H., Auclin E., Ni A., Martínez-Bernal G., Ferrara R., Lai W.V., Hendriks L.E.L. (2018). Impact of Baseline Steroids on Efficacy of Programmed Cell Death-1 and Programmed Death-Ligand 1 Blockade in Patients with Non-Small-Cell Lung Cancer. J. Clin. Oncol..

[20] Su J., Li Y., Tan S., Cheng T., Luo Y., Zhang L. (2025). Pretreatment neutrophil-to-lymphocyte ratio is associated with immunotherapy efficacy in patients with advanced cancer: A systematic review and meta-analysis. Sci. Rep..

[21] Bartlett E.K., Flynn J.R., Panageas K.S., Ferraro R.A., Sta Cruz J.M., Postow M.A., Coit D.G., Ariyan C.E. (2020). High neutrophil-to-lymphocyte ratio (NLR) is associated with treatment failure and death in patients who have melanoma treated with PD-1 inhibitor monotherapy. Cancer.

[22] De Cicco P., Ercolano G., Ianaro A. (2020). The New Era of Cancer Immunotherapy: Targeting Myeloid-Derived Suppressor Cells to Overcome Immune Evasion. Front. Immunol..

[23] Matsumoto K., Yamamoto Y., Shiroyama T., Kuge T., Mori M., Tamiya M., Kinehara Y., Tamiya A., Suzuki H., Tobita S. (2024). Risk Stratification According to Baseline and Early Change in Neutrophil-to-Lymphocyte Ratio in Advanced Non-Small Cell Lung Cancer Treated with Chemoimmunotherapy: A Multicenter Real-World Study. Target. Oncol..

[24] Cortellini A., Ricciuti B., Borghaei H., Naqash A.R., D’Alessio A., Fulgenzi C.A.M., Addeo A., Banna G.L., Pinato D.J. (2022). Differential prognostic effect of systemic inflammation in patients with non-small cell lung cancer treated with immunotherapy or chemotherapy: A post hoc analysis of the phase 3 OAK trial. Cancer.

[25] Bagley S.J., Kothari S., Aggarwal C., Bauml J.M., Alley E.W., Evans T.L., Kosteva J.A., Ciunci C.A., Gabriel P.E., Thompson J.C. (2017). Pretreatment neutrophil-to-lymphocyte ratio as a marker of outcomes in nivolumab-treated patients with advanced non-small-cell lung cancer. Lung Cancer.

[26] Cho A., Kranawetter B., Untersteiner H., Khalaveh F., Dorfer C., Rössler K., Zöchbauer-Müller S., Gatterbauer B., Hochmair M.J., Frischer J.M. (2021). Neutrophil-to-Lymphocyte Ratio Is Superior to Other Leukocyte-Based Ratios as a Prognostic Predictor in Non-Small Cell Lung Cancer Patients with Radiosurgically Treated Brain Metastases Under Immunotherapy or Targeted Therapy. World Neurosurg..

[27] Li H., Wang W., Yang X., Lian J., Zhang S., Cao J., Zhang X., Song X., Jia S., Xue R. (2020). The Clinical Prognostic Value of the Neutrophil-to-Lymphocyte Ratio in Brain Metastases from Non-Small Cell Lung Cancer-Harboring EGFR Mutations. Cancer Manag. Res..

[28] Goldberg S.B., Gettinger S.N., Mahajan A., Chiang A.C., Herbst R.S., Sznol M., Tsiouris A.J., Cohen J., Vortmeyer A., Jilaveanu L. (2016). Pembrolizumab for patients with melanoma or non-small-cell lung cancer and untreated brain metastases: Early analysis of a non-randomised, open-label, phase 2 trial. Lancet Oncol..

[29] Slotman B., Faivre-Finn C., Kramer G., Rankin E., Snee M., Hatton M., Postmus P., Collette L., Musat E., Senan S. (2007). Prophylactic cranial irradiation in extensive small-cell lung cancer. N. Engl. J. Med..

[30] Takahashi T., Yamanaka T., Seto T., Harada H., Nokihara H., Saka H., Nishio M., Kaneda H., Takayama K., Ishimoto O. (2017). Prophylactic cranial irradiation versus observation in patients with extensive-disease small-cell lung cancer: A multicentre, randomised, open-label, phase 3 trial. Lancet Oncol..

[31] Soria J.C., Ohe Y., Vansteenkiste J., Reungwetwattana T., Chewaskulyong B., Lee K.H., Dechaphunkul A., Imamura F., Nogami N., Kurata T. (2018). Osimertinib in Untreated EGFR-Mutated Advanced Non-Small-Cell Lung Cancer. N. Engl. J. Med..

[32] Shi Y., Zhang L., Liu X., Zhou C., Zhang L., Zhang S., Wang D., Li Q., Qin S., Hu C. (2013). Icotinib versus gefitinib in previously treated advanced non-small-cell lung cancer (ICOGEN): A randomised, double-blind phase 3 non-inferiority trial. Lancet Oncol..

[33] Wang M., Yang J.C., Mitchell P.L., Fang J., Camidge D.R., Nian W., Chiu C.H., Zhou J., Zhao Y., Su W.C. (2022). Sunvozertinib, a Selective EGFR Inhibitor for Previously Treated Non-Small Cell Lung Cancer with EGFR Exon 20 Insertion Mutations. Cancer Discov..

[34] Gijtenbeek R.G.P., Damhuis R.A.M., van der Wekken A.J., Hendriks L.E.L., Groen H.J.M., van Geffen W.H. (2023). Overall survival in advanced epidermal growth factor receptor mutated non-small cell lung cancer using different tyrosine kinase inhibitors in The Netherlands: A retrospective, nationwide registry study. Lancet Reg. Health Eur..

[35] Masuda T., Tsubata Y., Hata K., Horie M., Kiura K., Kanaji N., Inoue T., Kodani M., Yanai M., Yamaguchi K. (2024). Efficacy of immune checkpoint inhibitors according to programmed cell death-ligand 1 expression in patients with non-small cell lung cancer and brain metastasis: A real-world prospective observational study. Thorac. Cancer.

[36] Liu B., Chen J., Luo M. (2025). Efficacy and safety of immune checkpoint inhibitors for brain metastases of non-small cell lung cancer: A systematic review and network meta-analysis. Front. Oncol..

[37] Park S., Baldry R., Jung H.A., Sun J.M., Lee S.H., Ahn J.S., Kim Y.J., Lee Y., Kim D.W., Kim S.W. (2024). Phase II Efficacy and Safety of 80 mg Osimertinib in Patients with Leptomeningeal Metastases Associated with Epidermal Growth Factor Receptor Mutation-Positive Non-Small Cell Lung Cancer (BLOSSOM). J. Clin. Oncol..

