# Supplementary material for: Alteration of Prognostic Factors for Patients with Brain Metastases from Lung Cancer Before and After the Introduction of Immune Checkpoint Inhibitors: A Retrospective Single-Institution Study
Source: Cancers (Basel). 2025 Sep 19;17(18):3067. doi: 10.3390/cancers17183067 (PMC12468549; doi:10.3390/cancers17183067)
Supplement: Supplementary file 1 [file cancers-17-03067-s001.zip › cancers-3802443-supplementary.pdf]

cancers

MDPI

Supplemental Table S1. Clinical characteristics, treatment, and survival outcomes of patients before and after the introduction of ICIs.

Patient_ ID	Sex	Age	NLR	post2019	Primary Pathological Diagnosis	ICIs	Molecular Targeted Therapy	OS (month)	OS Event
001	Female	83	8.2	(−)	adenocarcinoma	(−)	(−)	2.6	death
002	Male	71	2.9	(−)	squamous cell carcinoma	(−)	(−)	5.6	death
003	Female	65	4.2	(−)	squamous cell carcinoma	(−)	(−)	3.0	death
004	Male	64	1.5	(−)	small cell carcinoma	(−)	(−)	4.7	death
005	Male	65	1.7	(−)	adenocarcinoma	(−)	(+)	58.0	death
006	Male	76	2.1	(−)	adenocarcinoma	(−)	(+)	29.3	death
007	Male	81	2.7	(−)	small cell carcinoma	(−)	(−)	3.3	death
008	Female	69	10.4	(−)	squamous cell carcinoma	(−)	(−)	2.8	death
009	Female	72	12.0	(−)	adenocarcinoma	(−)	(+)	3.3	death
010	Male	64	12.1	(−)	adenocarcinoma	(−)	(+)	15.9	death
011	Male	82	2.2	(−)	squamous cell carcinoma	(−)	(−)	15.4	death
012	Male	68	3.0	(−)	squamous cell carcinoma	(−)	(−)	14.0	death
013	Male	64	3.7	(−)	adenocarcinoma	(−)	(−)	0.6	death
014	Male	69	3.0	(−)	small cell carcinoma	(−)	(−)	7.4	death
015	Male	65	3.5	(−)	adenocarcinoma	(−)	(+)	1.4	death
016	Male	78	6.5	(−)	adenocarcinoma	(−)	(−)	4.3	death
017	Male	78	11.8	(−)	adenocarcinoma	(−)	(+)	3.9	death
018	Male	83	3.9	(−)	small cell carcinoma	(−)	(+)	3.4	death
019	Male	65	2.9	(−)	small cell carcinoma	(−)	(+)	3.4	death
020	Female	76	1.2	(−)	adenocarcinoma	(−)	(+)	18.4	death
021	Male	92	4.4	(−)	small cell carcinoma	(−)	(−)	3.5	death
022	Male	79	2.5	(−)	squamous cell carcinoma	(−)	(−)	7.0	death
023	Male	63	2.2	(−)	squamous cell carcinoma	(−)	(−)	1.7	death
024	Female	79	3.0	(−)	adenocarcinoma	(−)	(+)	10.2	death
025	Female	64	4.8	(−)	adenocarcinoma	(−)	(+)	109.9	censored
026	Female	54	2.9	(−)	squamous cell carcinoma	(−)	(−)	6.1	death
027	Female	94	3.7	(−)	adenocarcinoma	(−)	(−)	8.1	death
028	Female	65	1.8	(−)	adenocarcinoma	(−)	(+)	10.0	death
029	Female	77	1.6	(−)	adenocarcinoma	(−)	(+)	19.8	death
030	Male	73	6.1	(−)	squamous cell carcinoma	(−)	(−)	2.3	death
031	Male	85	9.7	(−)	adenocarcinoma	(−)	(−)	1.4	death
032	Male	73	2.8	(−)	small cell carcinoma	(−)	(−)	19.2	death
033	Male	56	8.2	(−)	adenocarcinoma	(+)	(−)	84.4	death
034	Male	87	3.5	(−)	squamous cell carcinoma	(−)	(−)	1.7	death
035	Male	48	6.4	(−)	small cell carcinoma	(−)	(−)	10.9	death

036	Male	54	6.6	(−)	adenocarcinoma	(−)	(−)	1.9	death
037	Male	64	1.9	(−)	small cell carcinoma	(−)	(−)	3.5	death
038	Female	55	6.9	(−)	adenocarcinoma	(−)	(+)	46.3	death
039	Male	74	2.6	(−)	adenocarcinoma	(−)	(+)	6.7	death
040	Female	70	6.2	(−)	adenocarcinoma	(−)	(+)	0.3	death
041	Female	69	4.0	(−)	adenocarcinoma	(−)	(+)	7.8	death
042	Male	78	2.7	(−)	small cell carcinoma	(−)	(−)	6.7	death
043	Male	70	6.1	(−)	adenocarcinoma	(−)	(−)	1.7	death
044	Male	78	2.0	(−)	small cell carcinoma	(−)	(−)	0.8	death
045	Female	75	5.6	(−)	squamous cell carcinoma	(−)	(−)	2.2	death
046	Female	75	4.4	(−)	squamous cell carcinoma	(−)	(−)	7.7	death
047	Male	70	2.1	(−)	adenocarcinoma	(−)	(+)	0.7	death
048	Male	82	11.6	(−)	adenocarcinoma	(−)	(−)	2.2	death
049	Male	69	1.5	(−)	adenocarcinoma	(−)	(+)	3.5	death
050	Male	68	5.3	(−)	adenocarcinoma	(−)	(+)	9.8	death
051	Male	76	1.8	(−)	squamous cell carcinoma	(−)	(−)	3.1	death
052	Female	49	5.0	(−)	adenocarcinoma	(−)	(+)	14.7	death
053	Female	73	6.2	(−)	adenocarcinoma	(−)	(+)	6.8	death
054	Male	67	2.2	(−)	adenocarcinoma	(−)	(+)	1.0	death
055	Male	74	2.6	(−)	adenocarcinoma	(−)	(+)	14.1	death
056	Male	90	0.8	(−)	adenocarcinoma	(−)	(−)	14.5	death
057	Male	74	3.1	(−)	small cell carcinoma	(−)	(−)	1.3	death
058	Female	69	3.4	(−)	adenocarcinoma	(−)	(−)	1.9	death
059	Male	69	2.9	(−)	small cell carcinoma	(−)	(−)	4.2	death
060	Male	58	4.6	(−)	adenocarcinoma	(−)	(−)	1.7	death
061	Male	68	4.9	(−)	adenocarcinoma	(−)	(+)	5.9	death
062	Female	96	4.7	(−)	squamous cell carcinoma	(−)	(−)	4.6	death
063	Male	75	6.0	(−)	adenocarcinoma	(−)	(−)	1.3	death
064	Male	85	9.0	(−)	squamous cell carcinoma	(−)	(−)	1.5	death
065	Female	52	3.0	(−)	adenocarcinoma	(−)	(+)	3.4	death
066	Female	77	26.0	(−)	adenocarcinoma	(−)	(+)	16.6	death
067	Male	77	4.5	(−)	adenocarcinoma	(−)	(+)	26.5	death
068	Female	70	1.7	(−)	adenocarcinoma	(−)	(−)	4.8	death
069	Male	70	13.0	(−)	adenocarcinoma	(−)	(−)	1.7	death
070	Female	66	2.6	(−)	adenocarcinoma	(−)	(+)	20.2	death
071	Female	88	6.3	(−)	adenocarcinoma	(−)	(+)	3.4	death
072	Female	76	1.9	(−)	adenocarcinoma	(−)	(−)	4.8	death
073	Male	73	9.0	(−)	small cell carcinoma	(−)	(−)	7.7	death
074	Male	71	1.5	(−)	adenocarcinoma	(−)	(+)	3.5	death
075	Male	73	10.0	(−)	adenocarcinoma	(−)	(−)	1.0	death
076	Male	69	4.3	(−)	small cell carcinoma	(−)	(−)	1.8	death
077	Male	63	15.0	(−)	small cell carcinoma	(−)	(−)	2.8	death
078	Male	79	4.2	(−)	squamous cell carcinoma	(+)	(−)	1.8	death
079	Male	66	4.3	(−)	adenocarcinoma	(−)	(−)	7.5	death
080	Male	60	4.6	(−)	adenocarcinoma	(−)	(+)	24.9	death
081	Male	67	2.9	(−)	adenocarcinoma	(−)	(−)	5.0	death
082	Male	82	3.3	(−)	adenocarcinoma	(−)	(−)	3.1	death

083	Male	60	2.5	(−)	adenocarcinoma	(−)	(+)	4.9	death
084	Female	86	2.3	(−)	adenocarcinoma	(−)	(−)	3.7	death
085	Male	86	5.4	(−)	small cell carcinoma	(−)	(−)	9.5	death
086	Female	51	3.4	(−)	adenocarcinoma	(−)	(+)	61.7	death
087	Male	73	3.5	(−)	adenocarcinoma	(−)	(−)	4.9	death
088	Male	67	2.5	(−)	adenocarcinoma	(−)	(−)	8.3	death
089	Male	68	6.6	(−)	adenocarcinoma	(−)	(+)	27.5	death
090	Male	70	7.2	(−)	adenocarcinoma	(−)	(−)	24.2	death
091	Male	89	3.9	(−)	squamous cell carcinoma	(−)	(−)	1.6	death
092	Female	77	5.5	(−)	adenocarcinoma	(−)	(+)	9.5	death
093	Female	85	3.9	(−)	adenocarcinoma	(−)	(+)	23.2	death
094	Male	77	3.2	(+)	small cell carcinoma	(−)	(−)	2.6	death
095	Female	54	5.7	(+)	adenocarcinoma	(+)	(−)	35.7	death
096	Female	74	2.6	(+)	adenocarcinoma	(−)	(−)	4.0	death
097	Male	57	3.1	(+)	adenocarcinoma	(−)	(+)	0.5	death
098	Male	65	9.6	(+)	adenocarcinoma	(−)	(+)	25.9	death
099	Male	74	9.6	(+)	adenocarcinoma	(−)	(−)	0.8	death
100	Female	70	4.1	(+)	adenocarcinoma	(+)	(−)	8.6	death
101	Male	68	1.3	(+)	adenocarcinoma	(+)	(−)	8.5	death
102	Male	65	1.8	(+)	adenocarcinoma	(−)	(−)	0.1	death
103	Female	73	1.7	(+)	adenocarcinoma	(−)	(−)	37.2	death
104	Female	79	12.5	(+)	squamous cell carcinoma	(−)	(−)	7.3	death
105	Male	82	3.1	(+)	small cell carcinoma	(−)	(−)	2.6	death
106	Female	75	14.8	(+)	adenocarcinoma	(+)	(+)	1.3	death
107	Female	79	2.6	(+)	adenocarcinoma	(−)	(+)	72.7	death
108	Female	91	3.7	(+)	adenocarcinoma	(−)	(+)	2.7	death
109	Female	71	3.0	(+)	adenocarcinoma	(+)	(+)	52.1	death
110	Male	83	3.9	(+)	squamous cell carcinoma	(−)	(−)	8.0	death
111	Male	69	4.0	(+)	adenocarcinoma	(−)	(+)	1.4	death
112	Male	79	3.4	(+)	squamous cell carcinoma	(−)	(−)	7.7	death
113	Male	84	1.0	(+)	adenocarcinoma	(−)	(−)	27.3	death
114	Male	73	4.4	(+)	squamous cell carcinoma	(−)	(−)	13.5	death
115	Female	73	6.2	(+)	adenocarcinoma	(−)	(−)	6.8	death
116	Male	69	4.2	(+)	adenocarcinoma	(−)	(+)	17.4	death
117	Female	92	2.9	(+)	squamous cell carcinoma	(−)	(−)	6.3	death
118	Female	80	3.0	(+)	adenocarcinoma	(−)	(+)	28.9	death
119	Female	73	4.1	(+)	adenocarcinoma	(−)	(+)	26.1	death
120	Male	90	8.5	(+)	adenocarcinoma	(−)	(−)	3.2	death
121	Male	77	3.7	(+)	adenocarcinoma	(−)	(+)	4.0	death
122	Female	74	6.6	(+)	adenocarcinoma	(−)	(−)	1.9	death
123	Male	73	0.9	(+)	small cell carcinoma	(−)	(+)	35.9	death
124	Male	79	6.0	(+)	small cell carcinoma	(−)	(−)	2.4	death
125	Male	82	34.0	(+)	adenocarcinoma	(−)	(+)	5.6	death
126	Female	59	4.6	(+)	adenocarcinoma	(+)	(+)	1.0	death
127	Male	65	0.7	(+)	small cell carcinoma	(−)	(+)	13.3	death
128	Male	87	3.9	(+)	squamous cell carcinoma	(−)	(−)	2.8	death

129	Female	69	5.9	(+)	adenocarcinoma	(−)	(+)	4.0	death
130	Male	69	6.7	(+)	adenocarcinoma	(−)	(+)	4.9	death
131	Male	68	3.8	(+)	squamous cell carcinoma	(+)	(−)	43.8	censored
132	Male	71	8.6	(+)	small cell carcinoma	(+)	(−)	15.8	death
133	Female	81	1.9	(+)	adenocarcinoma	(−)	(+)	23.4	death
134	Male	66	3.4	(+)	adenocarcinoma	(−)	(+)	25.9	death
135	Female	74	2.6	(+)	adenocarcinoma	(−)	(+)	76.2	death
136	Female	77	0.8	(+)	other lung carcinoma	(−)	(+)	60.9	censored
137	Male	82	11.2	(+)	squamous cell carcinoma	(−)	(−)	1.3	death
138	Female	50	3.4	(+)	adenocarcinoma	(−)	(+)	50.4	death
139	Male	74	2.9	(+)	small cell carcinoma	(+)	(−)	23.8	death
140	Male	68	3.8	(+)	squamous cell carcinoma	(+)	(−)	43.3	censored
141	Male	60	11.3	(+)	small cell carcinoma	(+)	(−)	2.6	death
142	Male	81	3.3	(+)	adenocarcinoma	(−)	(−)	1.8	death
143	Female	76	6.7	(+)	adenocarcinoma	(−)	(+)	24.6	death
144	Male	67	2.4	(+)	adenocarcinoma	(+)	(+)	62.5	death
145	Male	82	28.5	(+)	squamous cell carcinoma	(−)	(−)	1.3	death
146	Male	59	2.8	(+)	adenocarcinoma	(+)	(−)	26.5	censored
147	Male	48	2.8	(+)	adenocarcinoma	(−)	(−)	10.6	death
148	Male	64	0.6	(+)	adenocarcinoma	(−)	(+)	4.9	death
149	Male	92	2.5	(+)	squamous cell carcinoma	(−)	(−)	7.1	death
150	Male	79	1.8	(+)	squamous cell carcinoma	(+)	(−)	24.4	censored
151	Male	82	2.7	(+)	small cell carcinoma	(−)	(−)	21.3	censored
152	Female	89	2.3	(+)	squamous cell carcinoma	(−)	(−)	3.9	death
153	Female	59	3.4	(+)	adenocarcinoma	(−)	(−)	21.0	censored
154	Male	85	4.5	(+)	adenocarcinoma	(−)	(−)	22.2	censored
155	Female	77	0.5	(+)	small cell carcinoma	(−)	(−)	3.3	death
156	Female	74	5.5	(+)	adenocarcinoma	(+)	(−)	12.5	death
157	Female	53	10.2	(+)	adenocarcinoma	(+)	(−)	31.4	censored
158	Male	74	10.8	(+)	small cell carcinoma	(−)	(−)	18.2	death
159	Female	64	7.0	(+)	small cell carcinoma	(−)	(−)	8.0	death
160	Male	70	3.5	(+)	squamous cell carcinoma	(+)	(−)	28.8	censored
161	Male	85	3.1	(+)	adenocarcinoma	(+)	(−)	9.5	death
162	Male	81	2.5	(+)	small cell carcinoma	(−)	(−)	11.0	death
163	Female	71	5.0	(+)	adenocarcinoma	(−)	(+)	24.9	censored
164	Male	61	6.0	(+)	adenocarcinoma	(+)	(+)	21.0	censored
165	Female	72	15.6	(+)	adenocarcinoma	(−)	(−)	2.3	death
166	Female	70	2.2	(+)	adenocarcinoma	(+)	(−)	9.7	death
167	Female	86	3.4	(+)	adenocarcinoma	(−)	(+)	23.0	censored
168	Female	72	2.4	(+)	small cell carcinoma	(−)	(−)	10.8	death
169	Female	81	2.1	(+)	adenocarcinoma	(−)	(+)	11.9	censored
170	Male	76	1.3	(+)	small cell carcinoma	(+)	(−)	15.0	censored
171	Male	78	1.4	(+)	squamous cell carcinoma	(−)	(−)	16.8	death
172	Male	57	2.8	(+)	adenocarcinoma	(−)	(−)	13.0	death

173	Female	55	1.6	(+)	adenocarcinoma	(−)	(+)	9.1	death
174	Female	81	4.1	(+)	squamous cell carcinoma	(−)	(−)	1.4	death
175	Male	70	1.9	(+)	adenocarcinoma	(+)	(−)	7.9	censored
176	Male	73	1.2	(+)	adenocarcinoma	(−)	(+)	7.8	censored
177	Male	75	5.0	(+)	squamous cell carcinoma	(+)	(−)	2.5	death
178	Female	74	7.2	(+)	adenocarcinoma	(+)	(−)	3.1	death
179	Female	75	1.0	(+)	adenocarcinoma	(−)	(+)	57.1	censored
180	Female	55	6.8	(+)	adenocarcinoma	(+)	(−)	8.6	death
181	Male	74	2.4	(+)	adenocarcinoma	(+)	(−)	7.7	censored
182	Male	74	3.1	(+)	adenocarcinoma	(−)	(+)	8.1	censored
183	Female	68	2.5	(+)	small cell carcinoma	(+)	(−)	9.3	censored
184	Male	78	2.8	(+)	small cell carcinoma	(−)	(−)	3.6	death
185	Male	82	2.9	(+)	adenocarcinoma	(−)	(−)	8.6	censored
186	Female	79	2.2	(+)	adenocarcinoma	(+)	(−)	6.7	censored

ICIs=immune checkpoint inhibitors, NLR=Neutrophile- lymphocyte ratio, OS=overall survival.

Supplementary Table S2. clinical characteristics of PreICIs (2014–2018) and PostICIs (2019–2023), excluding small cell carcinoma.

	Main Factor	Sub Factor	PreICIs	PostICIs	*p* Value
Pretreatment factors	age	over 70	45	52	0.31
		under 70	31	24	
	sex	female	30	36	0.41
		male	46	40	
	symptomatology	asymptomatic	38	39	1
		symptomatic	38	37	
	KPS	under 60	35	44	0.19
		over 70	41	32	
	cyst lesion	(−)	54	50	0.6
		(+)	22	26	
	hematoma	(−)	74	75	1
		(+)	2	1	
	meningitis	(−)	71	64	0.1
		(+)	5	12	
	extra cranial metastasis	(−)	45	43	0.87
		(+)	31	33	
	Charlson-Deyo score	0–1	56	63	0.24
		over 2	20	13	
	Number of metastatic lesions	multiple	52	46	0.40
		single	24	30	
	NLR<4	(−)	38	46	0.25
		(+)	38	30	
Therapeutic factors	surgery	(−)	67	64	0.64
		(+)	9	12	
	chemotherapy	(−)	14	12	0.83
		(+)	62	64	
	conventional chemotherapy	(−)	50	63	0.02 *
		(+)	26	13	
	Molecular targeted therapy	(−)	41	45	0.62

	(+)	35	31	
ICIs	(−)	74	53	<0.01 *
	(+)	2	23	
radiation	(−)	20	24	0.59
	(+)	56	52	
SRS	(−)	51	33	<0.01 *
	(+)	25	43	
whole brain radiation	(−)	42	66	<0.01 *
	(+)	34	10	
neuro death	(−)	67	42	0.10
	(+)	8	13	

ICI = immune checkpoint inhibitor, KPS = Karnofsky performance status, NLR = Neutrophil/Lymphocyte ratio, SRS = stereotactic radiosurgery, * = $p < 0.05$.

Supplementary Table S3**.** Multivariate analysis of median survival times for brain metastasis patients treated in pre-ICIs and post-ICIs, excluding small cell carcinoma.

PreICIs				PostICIs			
Factor	HR	95%CI	*p* Value	factor	HR	95%CI	*p* Value
KPS over70	0.53	0.33-0.87	0.01 *	KPS over70	0.47	0.33-0.67	<0.01 *
surgery	0.44	0.20-0.97	0.04 *	single lesion	0.69	0.49-0.99	0.04
Target chomotherapy	0.55	0.34-0.89	0.01 *	SRS	0.57	0.40-0.81	<0.01 *
				Target chomotherapy	0.51	0.36-0.74	<0.01 *
				ICIs	0.37	0.22-0.64	<0.01 *

CI = confidence interval, HR=hazard ratio, ICIs = immune checkpoint inhibitor KPS = karnofsky performance status, SRS=stereotactic radiosurgery, * = $p < 0.05$.

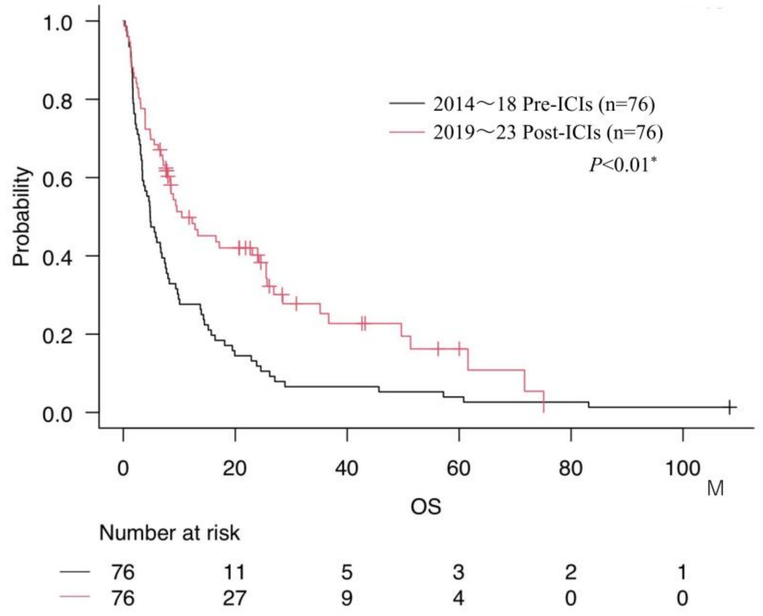

Supplemental Figure S1**.** Comparison of overall survival (mOS) between the Pre-ICIs and Post-ICIs groups, excluding patients with small cell carcinoma. * = $p < 0.05$.

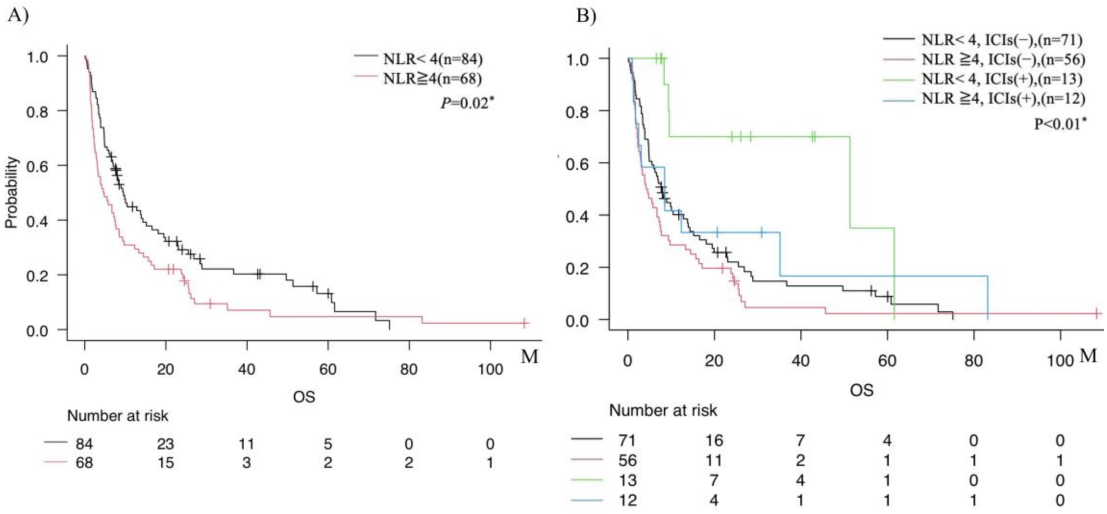

Supplemental Figure 2. (**A**) Comparison of overall survival between two groups dichotomized by NLR cutoff (NLR < 4 vs. NLR ≥ 4), excluding patients with small cell carcinoma. (**B**) Overall survival among four subgroups based on both NLR (cutoff 4) and ICI administration status (NLR < 4 with ICIs, NLR < 4 without ICIs, NLR ≥ 4 with ICIs, NLR ≥ 4 without ICIs), excluding patients with small cell carcinoma. * = $p < 0.05$.
